# Feasibility pilot study of the use of ultra-low dose iodinated contrast agent for endovascular procedures in patients with chronic limb-threatening ischemia and renal impairment: the ULTRA-LOW study

**DOI:** 10.1186/s42155-026-00725-4

**Published:** 2026-07-03

**Authors:** Stavros Spiliopoulos, Stavros Grigoriadis, Nikolaos Galanakis, Elias Kehagias, Konstantinos Palialexis, Ornella Moschovaki-Zeiger, Athanasios Giannakis, Ioannis Giannikouris, Petros Nikolopoulos, Constantine N. Antonopoulos, George Sfyroeras, Konstantinos Moulakakis, Andreas Lazaris, John Kakisis

**Affiliations:** 1 2nd Department of Radiology, Interventional Radiology Unit, Medical School, National and Kapodistrian University of Athens, Attikon” University General Hospital, Athens, Greece; 2 Department of Radiology, Medical School, University Hospital of Heraklion, University of Crete, Heraklion, Greece; 3 Department of Nephrology and Hemodialysis Unit, Mediterraneo Hospital, Glyfada, Athens, Attika Greece; 4 Clinical and Interventional Nephrology Unit, Attikon University General Hospital, Chaidari, Attikí 12462 Greece; 5 1st Vascular Surgery Department, School of Medicine, National and Kapodistrian University of Athens, Attikon” University Hospital, Athens, Greece

**Keywords:** Contrast-associated acute kidney injury, Chronic limb-threatening ischemia, Peripheral arterial endovascular interventions, Angioplasty, Iodinated contrast medium

## Abstract

**Purpose:**

To evaluate the feasibility and safety of an ultra-low-dose iodinated contrast media (CM) protocol during peripheral endovascular procedures in patients with chronic kidney disease (CKD) and chronic limb-threatening ischemia (CLTI).

**Materials and methods:**

This prospective, two-center, observational study included consecutive patients with CLTI and renal dysfunction (eGFR < 60 mL/min/1.73m^2^, stages 2–4) undergoing infrainguinal revascularization over a 1-year period. The protocol utilized 1:9 or 2:8 CM-to-saline dilutions to minimize iodine exposure. Primary endpoints were technical success (revascularization using ≤ 15 mL CM) and the incidence of Contrast-Associated Acute Kidney Injury (CI-AKI), defined as a 25% increase from baseline or a 0.5 mg/dL increase in absolute sCr value, within 72 h. Secondary endpoints included procedural success, limb salvage, and freedom from clinically driven target lesion revascularization (TLR) at 6 months.

**Results:**

Eighteen patients were enrolled, presenting advanced (Rutherford 6: 38.9%; occlusions: 38.8%) and complex infrainguinal disease (both femoropopliteal and infrapopliteal disease 27.7%; occlusions 38.8%). Technical success was 94.5%, with a mean CM volume of 10.3 ± 3.5 mL (range: 5–17 mL). Procedural success was 100%. No cases of CI-AKI occurred; one patient (5.5%) required dialysis 3 months post-procedure due to disease progression. Mean sCr and eGFR significantly improved at 72 h compared to baseline (p < 0.0001). At 6 months, limb salvage was 94.4%, TLR-free rate was 81.3% and survival was 83.3%.

**Conclusion:**

Ultra-low-dose iodinated CM protocol in complex CLTI endovascular treatment of CKD patients is safe and effective, achieving high technical success without evidence of renal function deterioration.

**Level of evidence:**

Level 4, Case Series.

## Introduction

Contrast-Associated Acute Kidney Injury (CI-AKI), historically named contrast-induced nephropathy (CIN), is a main cause of hospital-acquired acute kidney injury (AKI) and is associated with a significantly higher risk of in-hospital and 1-year mortality, even in patients eventually not requiring dialysis [1]. Notably, over 20% of patients requiring in-hospital dialysis due to CI-AKI eventually progress to Stage 5 end-stage renal disease (ESRD) [2]. Key risk factors include advanced age, peripheral arterial disease (PAD), dehydration, high contrast media (CM) volume or osmolarity, and nephrotoxic medications (e.g., ACE inhibitors, diuretics, NSAIDs). However, diabetes mellitus (DM) remains the primary risk factor; CI-AKI incidence in diabetic patients can reach 25%, with 10–15% requiring dialysis, compared to only 1–3% in non-diabetic subjects [2].

CI-AKI is defined as an acute impairment of kidney function—characterized by a 25% increase in serum creatinine (sCr) from baseline or a 0.5 mg/dL (44 µmol/L) increase in absolute sCr value, within 48–72 h after intravenous contrast administration (or intra-arterial CM administration with second-pass renal exposure) provided no other identifiable cause of kidney failure is present [2–6]. CI-AKI is typically transient with sCr levels usually normalizing within 14 days. Pre-existing chronic kidney disease (CKD) is a major risk factor. Consequently, international guidelines recommend aggressive hydration, the use of low-osmolarity CM, and strict minimization of contrast volume for patients with an eGFR < 60 mL/min/1.73m^2^ [7].

Percutaneous, endovascular treatment is a well-established minimally invasive treatment for the management of chronic limb-threatening ischemia (CLTI), offering faster recovery and lower complication rates than open surgery [8–11]. However, a significant proportion of CLTI patients present with comorbid DM and impaired renal function. To reduce CI-AKI risk, guidelines suggest intravenous hydration, CM dose limitation, use of low-osmolality CM and alternative agents such as CO_2_ or paramagnetic substances [7, 12]. Despite recommendations to limit iodinated CM in patients with eGFR < 60 mL/min/1.73m^2^, the specific "safe" dose threshold remains poorly defined. Standard practice typically utilizes dilutions ranging from 50 to 100% contrast, with total volumes often reaching 100–130 mL [5]. While CO_2_ angiography is an alternative, it often requires supplemental iodinated CM; a large 2015 multicenter registry reported an average adjunct CM volume of 15 mL and a 2% CO_2_-related mortality rate [13].

Advances in modern angiographic equipment have significantly enhanced image quality while simultaneously reducing radiation exposure. These technical improvements potentially allow for substantially lower CM doses during endovascular procedures. This study aims to investigate the feasibility and safety of an ultra-low-dose iodinated contrast protocol for endovascular procedures in high-risk patients with CLTI and renal impairment, utilizing a latest-generation angiography platform.

## Materials and methods

### Study design

The study was approved by the Hospitals’ Scientific and Ethics Committee and was registered in a public database (clinicaltrias.gov number: blinded for review). This was a prospective, two-center, observational study of consecutive patients with CLTI and renal function impairment but not on renal replacement therapy (RRT), (eGFR < 60 ml/min/1.73m^2^, kidney disease stages 2–4), who were scheduled to undergo endovascular revascularization procedures of the lower limb arteries, between a 1-year period (September 1 st 2024 and August 31th 2025), at the Interventional Radiology Units of the two study centers (blinded for review). Both are large tertiary University Hospitals and high-volume vascular centers, using the same latest-generation angiography platform. Treatment strategy was decided in a vascular multidisciplinary team meeting (MDT) after reviewing all available relevant axial imaging (previous CTA or MRA), and recent (within 1 week) Duplex ultrasound (DUS). CT-angiography was omitted to avoid CI-AKI; non-contrast CT or MRI was performed only if deemed necessary. The last patient was enrolled on July 23rd 2025 and the study follow-up was completed January 23rd 2026. Patient demographics and details of the procedure were recorded. Inclusion criteria were: (i) patients with Rutherford 4 to 6 class CLTI, (ii) eGFR < 60 ml/min/1.73m^2^ at the day of the procedure, (iii) MDT decision to proceed to any endovascular revascularization without restriction in technique and device use, (iv) lesions located at the common femoral artery and distally, including superficial femoral, popliteal, infrapopliteal arteries and pedal arch, (v) no restriction in arterial lesion type (occlusion or stenosis), lesion length, or calcification grade, (vi) age ≥ 18 years. Exclusion criteria included: (i) patient already receiving or scheduled to receive RRT (kidney disease stage 5), (ii) uncorrectable coagulopathy (INR > 1.5, PLT < 50.000), (iv) acute limb ischemia, (v) history of severe allergy to contrast media, (vi) pregnancy.

### Procedure

#### Endovascular technique

**Fig. 1:**
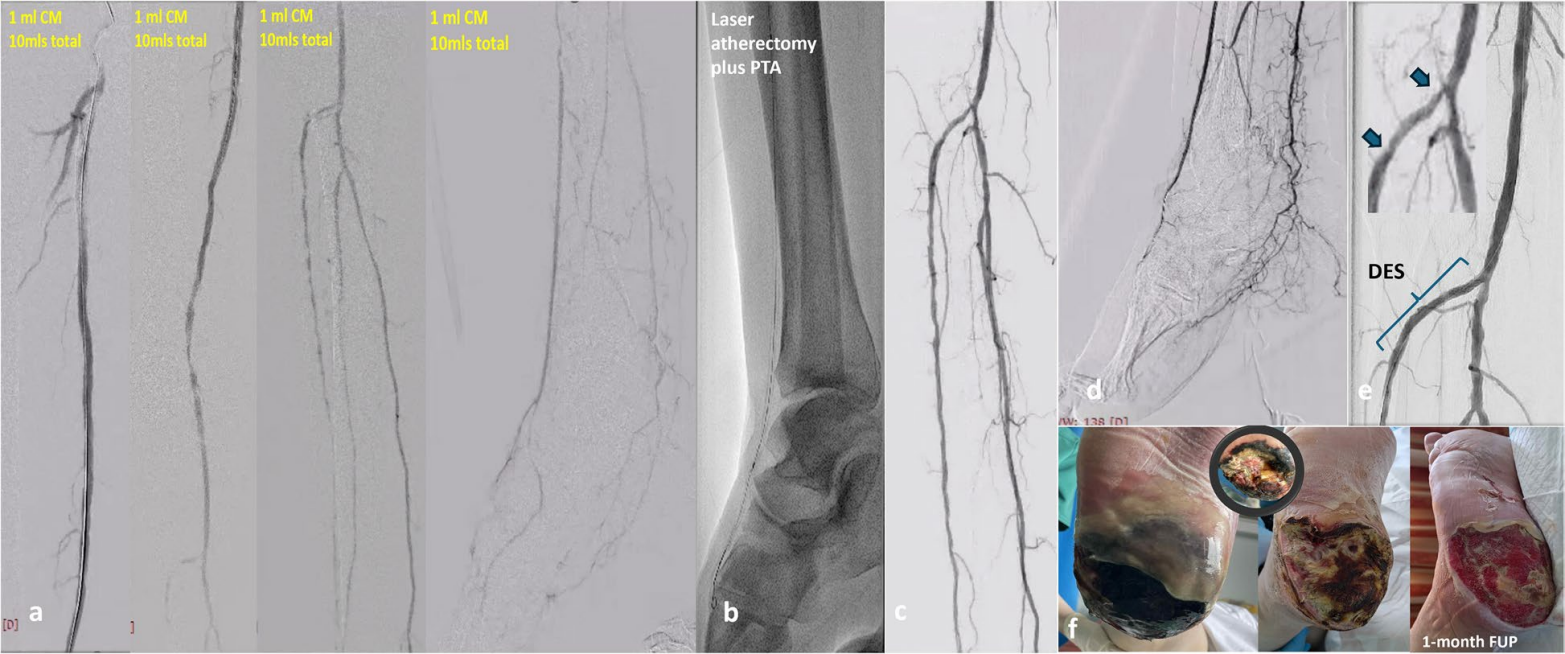
Infrapopliteal and pedal artery laser atherectomy. An 82-y–o male with IDDM presenting with Rutherford-Becker class 6 disease (infected gangrene and osteomyelitis). Baseline sCr: 2.49 and eGFR: 24. Pre-procedural DUS demonstrated both femoropopliteal and infrapopliteal disease. Antegrade CFA access was obtained. **a** Images of whole limb DSA using a total of 4mls CM (40mls 1:9 dilution), administrated from the 6Fr CFA sheath. **b** Representative fluoroscopic image of pedal laser atherectomy. **c** Final infrapopliteal DSA from an angiographic catheter at the level of P2, using 1 ml CN (10mls; 1:9 dilution) and (**d**) final DSA of the distal foot using again 1 ml of CM (10mls; 1:9 dilution), with magnified image (**e**) demonstrating outcome following DES placement (3.5 × 28 mm Albuminous +, Sirolimus -eluting stent) at the origin of the anterior tibial artery due to suboptimal angioplasty result (arrowheads). **f** Baseline, 7 days and 1 month follow up images of satisfactory wound healing following surgical debridement. Procedure performed with a total CM administration of 14mls

**Fig. 2:**
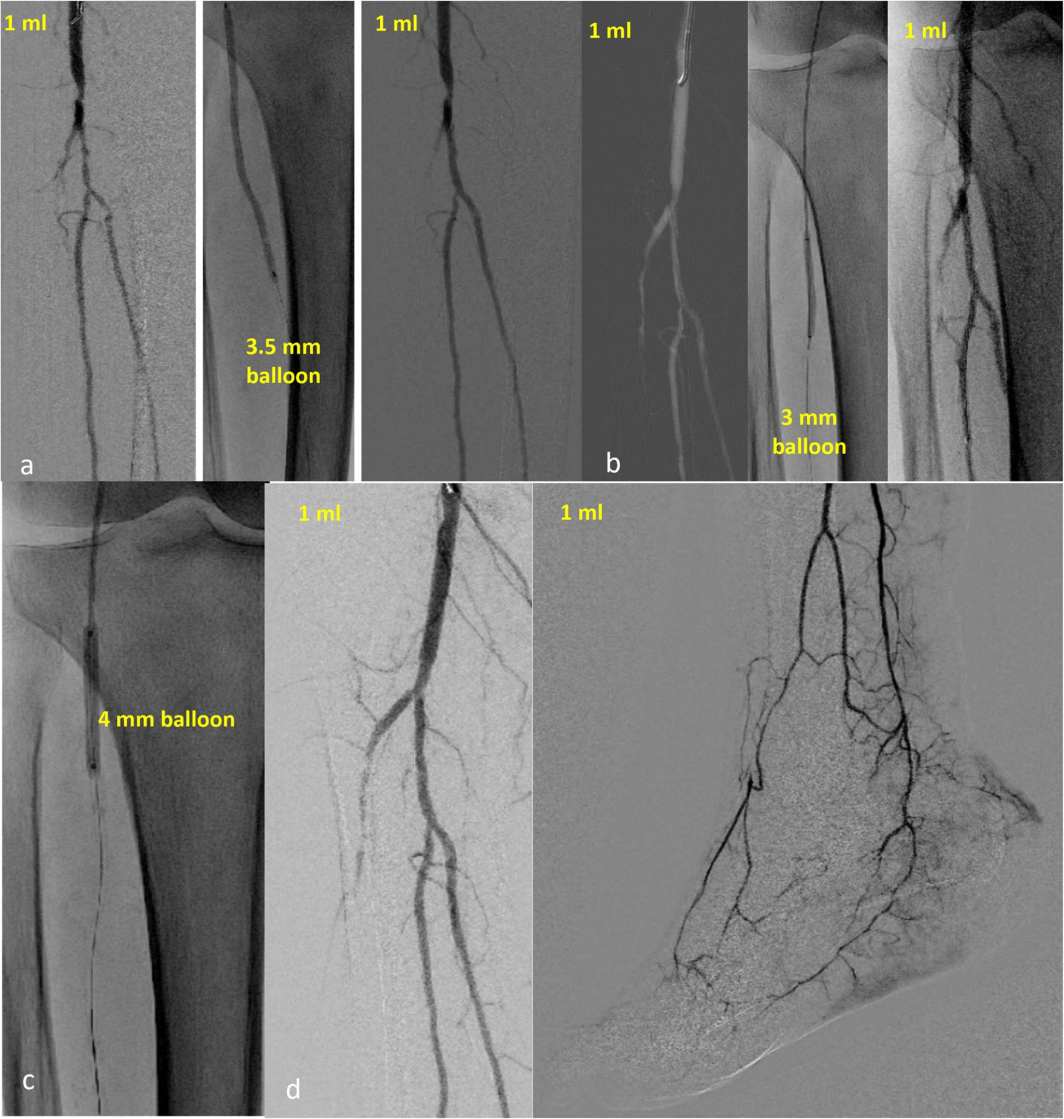
Step-by-stepinfrapoplitealbifurcation PTA. A 78-yo male patient with IDDM, presented with non-healing wound following 5th toe amputation due to wet gangrene. Pre-procedural DUS detected only infrapopliteal disease. Antegrade CFA access was obtained. **a** P3 and infrapopliteal DSA with the angiographic catheter at the level of P2, angioplasty of the P3, tibioperoneal and posterior tibial tandem stenosis with a 3,5 × 100 balloon (Coyote; Boston Scientific, USA), and post-angioplasty DSA using 3mls of CM (30mls; 1:9 dilution). **b** Roadmap image using 1 ml CM of the same dilution for the selective catheterization of the peroneal artery and 3 mm PTA of the peroneal artery stenosis Coyote; Boston Scientific, USA). Post-PTA DSA with the same amount/dilution and (**c**) additional 4 mm angioplasty (Coyote; Boston Scientific, USA) of the remaining P3 stenosis. **d** Final DSA of the infrapopliteal arteries and the distal foot (3). Procedure performed with a total 12mls of CM

**Fig. 3:**
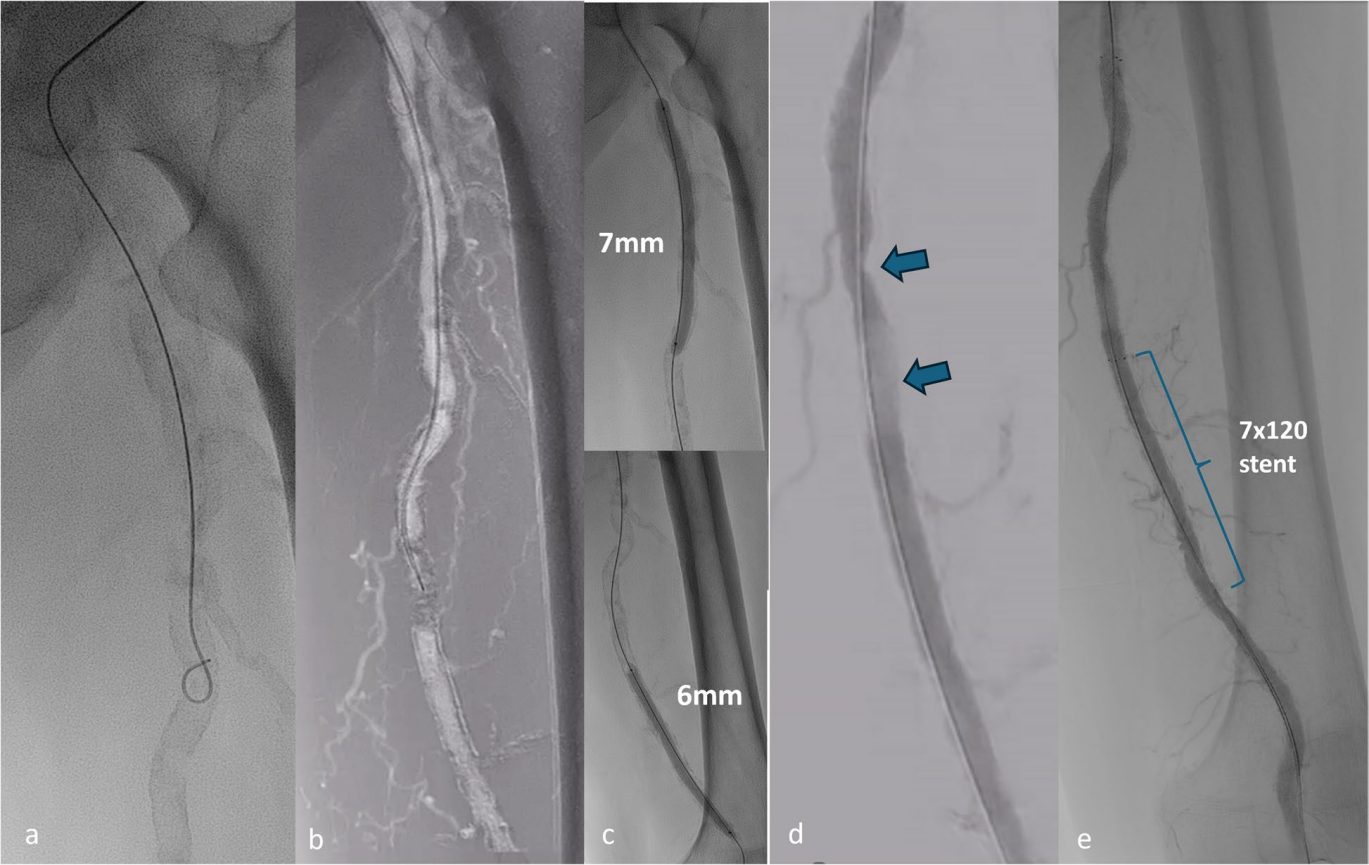
FemoropoplitealPTA and stenting. 65-year-old female transplant kidney patient presenting with Rutherford-Becker class 5 gangrene at 1 st to 3rd toe (sCr 1.7 and eGFR 39.6). Pre-procedural DUS detected and short 4 cm SFA occlusion and tandem SFA to P1 stenosis. **a** Initial fluoroscopic image following antegrade CFA access demonstrating SFA 360 calcifications of the target lesion. **b** Roadmap image of the SFA occlusion using 0.5 ml of CM (5mls, 1:9 dilution). **c** The lesion was crossed using the subintimal technique and balloon angioplasty was performed (Mustang, Boston Scientific; USA, 7 mm for the proximal SFA stenosis and 6 mm for distal SFA occlusion and P1 stenosis. **d** DSA following angioplasty demonstrated suboptimal result at the level of the occlusion and P1 segment. **e** Final DSA angiogram following the deployment of a 7 × 120 mm self-expandable stent (Lifestent; BD, USA). Procedure performed with a total CM administration of 5mls, including a final DSA of the distal foot

Written informed consent was obtained from all patients prior to the procedure. Endovascular interventions were performed in two interventional radiology units equipped with recently installed biplane angiography systems (Azurion 7 b20/15, Philips, The Netherlands). Procedures were conducted by five vascular interventional radiologists with 5 to 20 years of experience in peripheral arterial interventions. A 1:9 or 2:8 dilution of iso-osmotic iodinated contrast agent (Visipaque 320 mg/mL) with saline was utilized, adhering to the ALARA (As Low As Reasonably Achievable) principle. Antegrade common femoral artery (CFA) access was successfully obtained in all cases (18/18; 100%) using a 6Fr sheath. Initial standardized limb angiography was performed via hand injection, utilizing a maximum of 4 mL of contrast medium (CM): femoropopliteal imaging with two runs (0.5 mL each) and infrapopliteal/pedal arch imaging with three runs (totalling 3 mL; Fig. 1). For infrapopliteal revascularization, angiography was performed through a selective catheter positioned in the popliteal artery just proximal to the trifurcation; this approach enhanced visibility while further reducing total CM volume. Revascularization followed standard endovascular techniques—including intraluminal or subintimal crossing, balloon angioplasty, and stenting—consistent with international guidelines (Figs. 2 and 3). Procedural details are summarized in Table 1. Image acquisition parameters were identical to those used for patients with normal eGFR undergoing standard infrainguinal treatments.

**Table 1:** Baseline patient demographics and procedural details

Patients (n)	18
Limbs	18
Mean age (years)	73 ± 9 (58–88)
Diabetes mellitus	17/18 (94.4%)
Coronary disease	9/18 (50%)
Smoking	6/18 (33.3%)
Hyperlipidemia	18/18 (100.0%)
Hypertension	18/18 (100.0%)
Mean body mass index (range)	26.7 ± 5.2 kg/m^2^ (20.7- 37.8)
**CLTI stage**	
Rutherford 4	2/18 (11.1%)
Rutherford 5	9/18 (50.0%)
Rutherford 6	7/18 (38.9%)
**Calcifications:**	
None	0/18 (0.0%)
Mild	2/18 (11.1%)
Moderate	6/18 (33.3%)
Severe	10/18 (55.6%)
**Lesion location**	
Femoropopliteal only	10/18 (55.5%)
BTK only	3/18 (16.6%)
Femoropopliteal and BTK	5/18 (27.7%)
Pedal arch	3/18 (16.6%)
**Occlusions**	7/18 (38.8%)
Subintimal crossing	3/7 (42.8%)
**Endovascular treatment**	
Balloon angioplasty	18/18 (100.0%)
Stenting	4/18 (22.2%)
Atherectomy	1/18 (5.5%)

Continuous data are presented as mean ± standard deviation

Categorical data are presented as counts and percentages in the parentheses

#### Peri-procedural care and follow-up

Patients were hospitalized for at least 24 h before and 72 h after the procedure to ensure optimal management. The protocol included: (i) hydration using intravenous 0.9% normal saline (1 ml/kg/h) starting 6–12 h pre-procedure and continuing post-procedure, (ii) serial assessment (24 h, 48 h and 72 h) of serum creatinine (sCr) and eGFR to screen for CI-AKI and (iii) medication management (nephrotoxic medications were withheld or modified based on nephrology consultation).

Regular clinical follow-up was scheduled at 1- and 6-months post-procedure, and annually thereafter.

#### Endpoints and definitions

The primary endpoints of the study were the assessment of the rate of CI-AKI estimated by comparing the eGFR and sCr levels at the day of the procedure just prior the procedure (baseline), versus 24 h, 48 h and 72 h following the procedure. CI-AKI was defined as a 25% increase in sCr from baseline or a 0.5 mg/dL (44 µmol/L) increase in absolute sCr value, within 48–72 h after contrast administration. Technical success defined as a successful, uneventful target lesion revascularization (TLR) with < 30% residual stenosis, using ≤ 15 mL of iso-osmotic iodinated contrast media, based on the average volumes of iodinated CN used in previously published data of CO_2_ procedures. [13] Secondary endpoints were procedural success defined as the restoration of at least one straight line of perfusion to the distal foot limb salvage rate at 6 months defined as the absence of above the knee major amputation of the target limb, procedure-related complications defined according to the CIRSE classification system [14], the number of patients requiring temporary or chronic renal replacement therapy within 6 months from the index procedure, freedom from clinically driven target lesion revascularization (TLR) due to symptoms relapse, and overall patient survival at 6 months follow-up. The contrast-to-eGRF ratio (volume of CM used during the procedure/baseline eGFR) was also calculated.

#### Statistical analysis

Discrete variables are presented as frequencies and percentages. Continuous data are reported as means ± standard deviation for normally distributed variables or as medians with interquartile ranges (IQR) for non-normal distributions, as determined by the Shapiro–Wilk test. We compared normally distributed continuous data using the paired Student’s t-test, while non-parametric data were analyzed via the Mann–Whitney U test. Time-dependent clinical outcomes, including limb salvage, overall survival and TLR rates, were estimated using Kaplan–Meier analysis. sCr and eGFR data were analyzed using Repeated Measures Analysis of Variance (RM-ANOVA) to evaluate the change in serum creatinine levels across four time points (Baseline, 24 h, 48 h, and 72 h). The RM-ANOVA was selected to account for the dependency between observations within the same subject. Following a significant global test, post-hoc pairwise comparisons were performed using Paired t-tests. To control for the inflation of Type I error (false positives) resulting from multiple comparisons, the Bonferroni correction was applied. All statistical tests were two-tailed, and an adjusted p-value of < 0.05 was considered statistically significant. Analysis was conducted on the full cohort of 18 subjects (N = 18). Statistical analysis was performed using the IBM SPSS statistical software (vers.26).

## Results

Over the 1-year study period, all patients with renal impairment identified by the vascular MDTs at the two participating centers as candidates for infrainguinal percutaneous endovascular treatment were enrolled. A total of 18 consecutive patients with Stage 2 to 4 CKD were investigated, the majority of whom (17/18) suffered from DM. Detailed baseline demographic data are reported in Table 1. The mean CM volume used was 10.3 ± 3.5 mL (range 5–17 mL), with a median of 10 mL. At baseline (day of procedure), the mean sCr and eGFR values were 2.13 ± 0.5 mg/dL and 35.83 ± 11.38 mL/min/1.73m^2^, respectively. All procedures were performed with a contrast to eGFR ratio < 1 (mean 0.37). The cohort included patients with advanced CLTI, with 38.9% classified as Rutherford stage 6. Complex infrainguinal disease was prevalent: 27.7% had multi-level (femoropopliteal and infrapopliteal) disease, 55.6% exhibited severe calcifications, and 38.8% involved total occlusions. Multi-level disease was treated in a single session, following standard protocols for patients with normal renal function. Procedural success was 100% (18/18). Only one significant puncture-site hematoma (1/18; 5.5%) occurred; this was a grade 2 complication that required no further treatment or prolonged hospitalization. No other procedure-related complications were observed. All patients underwent balloon angioplasty, with bailout stenting required in four cases: two bare self-expandable SFA stents, one combination of SFA and infrapopliteal drug-eluting stenting (DES), and one isolated infrapopliteal DES. Subintimal crossing was utilized in 3/7 occlusions, with a re-entry device required in one instance. Procedural details are analytically reported in Table 2.

**Table 2:** Comparison of baseline serum creatinine and eGFR levels with post-procedure timepoints and associated p-values

Time Point	sCr (mg/dL)	eGFR	sCr Sig	eGFR Sig
Baseline	2.13 ± 0.55	35.83 ± 11.38	—	—
24 Hours	1.93 ± 0.56	41.44 ± 12.70	***p*** < 0.001	***p*** < 0.001
48 Hours	1.84 ± 0.50	42.94 ± 14.39	***p*** < 0.001	***p*** < 0.01
72 Hours	1.86 ± 0.54	43.39 ± 15.75	***p*** < 0.001	***p*** < 0.01

Technical success was achieved in 94.4% of cases (17/18). In the single remaining case, 17 mL of contrast was required due to a high complexity of the lesion, which involved simultaneous SFA occlusion and infrapopliteal revascularization.

**Fig. 4:**
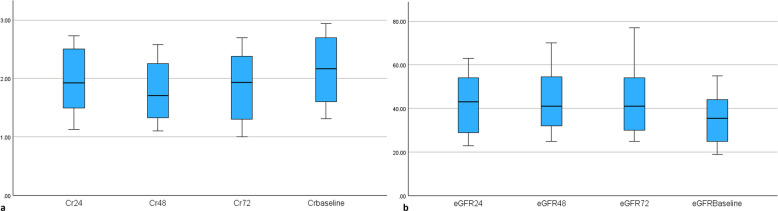
Box-plots representing (**a**) sCr and (**b**) eGFR values at baseline, 24 h, 48 h and 72 h

**Fig. 5:**
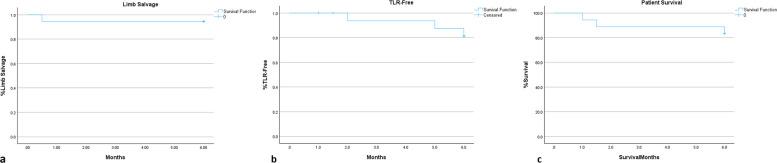
Kaplan–Meier estimates for (**a**) limb salvage, (**b**) TLR-free survival, and (**c**) overall patient survival over the 6-month follow-up period

There was a statistically significant decrease in sCr levels (p < 0.0001). Mean sCr decreased from a baseline of 2.13 ± 0.55 mg/dL to 1.93 ± 0.56 mg/dL at 24 h, 1.84 ± 0.50 mg/dL at 48 h, and 1.86 ± 0.54 mg/dL at 72 h. Post-hoc analysis revealed that the reduction from baseline was highly significant at all subsequent intervals (Table 2). No statistically significant differences were observed between the 24 h, 48 h, and 72 h time points (all p > 0.05), indicating that the primary reduction in creatinine occurred within the first 24 h and remained stable thereafter. Since eGFR and sCr are inversely related, the observed drop in sCr was accompanied by a corresponding significant increase in eGFR (Table 2). The changes in sCr and eGFR are represented in Fig. 4. At the 6-month follow-up, the limb salvage rate was 94.4%, the TLR-free survival rate was 81.3%, and the overall patient survival rate was 83.3% (Fig. 5).

## Discussion

This prospective pilot study demonstrates that the proposed ultra-low-dose iso-osmolar CM protocol is both safe and feasible for patients with stage 2 to 4 CKD undergoing infrainguinal endovascular revascularization for CLTI. The mean volume of CM administered was remarkably low at 10.3 ± 3.5 mL (range 5–17 mL). Notably, approximately 95% of the procedures (17/18 cases) were successfully completed using less than 15 mL of contrast media. As most infrainguinal interventions use 80–150 mL contrast, our ultra-low-dose protocol reduced contrast by roughly 90%. Additionally, the mean contrast-to-eGFR ratio was extremely low, further underscoring the minimal renal stress associated with the specific protocol [15]. There were no occurrences of CI-AKI within the study population. Renal replacement therapy (RRT) was required in only one patient (5.5%) during the 6-month follow-up period; however, this was not attributed to the index procedure. In this specific case, which utilized a total CM volume of 10 mL, RRT was initiated 3 months post-procedure due to underlying disease progression. This patient’s renal function actually showed transient improvement following the intervention, with eGFR increasing from a baseline of 22.4 mL/min/1.73m^2^ (sCr 2.8 mg/dL) to 24.5 mL/min/1.73m^2^ (sCr 2.57 mg/dL) at 72 h, remaining stable for over 1 month thereafter. Pre-existing CKD has been identified as a major risk factor for CI-AKI and current international guidelines suggest peri-procedural protocols to decrease the risk of CI-AKI in patients with eGFR < 60 ml/minute/1.73m^2^ [7, 12]. All patients in this study were hospitalized for a minimum of 4 days (at least 1 day pre-procedure and 3 days post-procedure) to facilitate drug therapy modification via nephrology consultation. This hospitalization period also ensured adequate peri-procedural hydration and rigorous monitoring of renal function to allow for immediate intervention should CI-AKI occur. Our data demonstrated an improvement in renal function manifested primarily in the first 24 h. sCr showed a sustained reduction, reaching its lowest point at 48 h. eGFR showed a corresponding sustained increase, reaching its peak at 72 h. The improvement in renal function observed during the first 72 h may partly reflect the effect of aggressive hydration and peri-procedural optimization rather than a direct renal protective effect of the low-contrast technique. The absence of a significant difference between the 24 h, 48 h, and 72 h intervals across both renal markers suggests that optimal pre-procedural pharmacotherapy modification and intravenous hydration achieve their maximum therapeutic impact early within the observation period. This is followed by a phase of physiological stabilization, during which the improved renal function is maintained. All endovascular procedures were performed following the ALARA principle for CM administration. The 0% incidence of CI-AKI reported in this cohort is consistent with the study’s design and optimized patient care. The authors hypothesized that modern angiographic systems maintain high image quality even with significantly diluted CM. This approach is physiologically sound; since CM exerts a direct toxic effect on renal tubular cells, reducing both the concentration and total volume minimizes renal exposure and mitigates the risk of acute tubular injury. Notably, infrainguinal peripheral endovascular procedures may inherently carry a lower risk of CI-AKI compared to more proximal interventions. This is largely because the contrast is injected distally, resulting in a "second-pass" exposure to the kidneys at a further reduced concentration compared to supra-renal or cardiac injections [12]. Notably, while the incidence of CI-AKI is traditionally reported to be higher following intra-arterial procedures compared to intravenous (IV) administration, contemporary data suggest a lower risk than previously assumed. A propensity-matched retrospective analysis of over 2,000 patients undergoing percutaneous coronary intervention (PCI) reported a CI-AKI incidence of only 2.3% [16]. This is considerably lower than the 5.0%–6.4% incidence rates following IV administration reported in two separate meta-analyses [17, 18]. This disparity can be explained by the hemodynamics of contrast delivery. During PCI, intra-arterial administration results in "first-pass" renal exposure, where the contrast media reaches the renal arteries in a relatively undiluted state. In contrast, during infrainguinal procedures, the CM reaches the kidneys only after entering systemic circulation and undergoing significant dilution—a "second pass" exposure that mirrors the physiological profile of IV administration. Consequently, the inherent risk of CI-AKI in infrainguinal interventions should be lower than that of more proximal arterial procedures [12].

The efficacy of this ultra-low CM protocol is demonstrated by a 100% procedural success rate and a high 6-month amputation-free survival rate. These results align with standard endovascular outcomes for similar CLTI populations without renal impairment [9, 19].

The efficacy of this ultra-low-dose CM protocol is underscored by a 100% procedural success rate and a high 6-month amputation-free survival rate. These clinical outcomes align with established endovascular benchmarks for CLTI populations without renal impairment [9, 19]. Notably, nearly one-third of the cases involved simultaneous femoropopliteal and infrapopliteal interventions. These procedures reflected real-world clinical complexity, including severe calcification, total occlusions, and multi-level infrapopliteal disease. Collectively, these findings suggest that aggressive CM dilution does not compromise the technical precision or imaging quality required for complex revascularization. This success is likely attributable to the high-resolution imaging capabilities of the latest-generation angiography systems used in this study. This is consistent with previous research; for instance, Jens et al. reported that utilizing a 140 mg/mL iodine concentration for infrainguinal PAD interventions can reduce the total iodine load by over 40% without compromising diagnostic or interventional image quality [5].

While international guidelines suggest non-iodinated alternatives such as CO_2_ and paramagnetic agents [7, 12], our ultra-low-dose protocol appears to offer a favorable safety and efficacy profile compared to existing CO_2_ data. Two key points merit consideration. First, CO_2_ is associated with potential severe adverse events. Fujihara et al. reported a 17.3% complication rate in a registry of 98 patients, including two deaths from nonocclusive mesenteric ischemia [10, 20]. Second, CO_2_ use often fails to completely eliminate the need for iodinated CM. A CI-AKI incidence as high as 6.1% has been reported in CO_2_ studies, likely due to the supplementary iodinated CM required to optimize visualization [21]. Furthermore, CO_2_ presents practical challenges, including patient discomfort from gas displacement and a significant learning curve for image interpretation [21]. Gadolinium-based contrast agents (GBCAs) have also been explored as alternatives, either alone or combined with CO_2._ However, clinical experience remains sparse, and unresolved challenges regarding standardized dosing and imaging protocols currently hinder the widespread adoption of GBCAs in routine practice [22].

The primary limitations of this study include the small sample size, which restricts the statistical power of the results, and the lack of a control group (per es, versus CO_2_) for direct comparison. Future randomized controlled trials are necessary to provide robust comparative data between these various imaging protocols in patients with renal impairment.

In conclusion, this ultra-low-dose iodinated CM protocol for infrainguinal procedures proved safe and effective, achieving a 0% CI-AKI rate alongside high technical and clinical success. These results suggest that by leveraging modern angiographic technology, clinicians can significantly reduce contrast exposure in high-risk CLTI patients without sacrificing procedural quality. Further comparative studies are warranted to validate these findings on a larger scale.

## Data Availability

The datasets used and/or analyzed during the current study are available from the corresponding author on reasonable request.
